# High 2-year mortality and recurrent infection rates after surgical treatment for primary septic arthritis of the hip in adult patients: An observational study

**DOI:** 10.1097/MD.0000000000016765

**Published:** 2019-08-09

**Authors:** Feng-Chen Kao, Yao-Chun Hsu, Pao-Hsin Liu, Yuan-Kun Tu, I-Ming Jou

**Affiliations:** a Department of Orthopedics, E-Da Dachang Hospital; b School of Medicine for International Students, College of Medicine, I-Shou University, Kaohsiung; c School of Medicine, Big Data Research Center, Fu-Jen Catholic University; d Division of Gastroenterology, Fu-Jen Catholic University Hospital, New Taipei; e Graduate Institute of Clinical Medicine, China Medical University, Taichung; f Division of Gastroenterology and Hepatology, E-Da Hospital; g Department of Biomedical Engineering, College of Medicine, I-Shou University, Kaohsiung, Taiwan.

**Keywords:** arthroplasty, mortality, septic hip, total-hip replacement

## Abstract

Primary septic arthritis of the hip is rare and potentially devastating in adults. Its optimal surgical treatment and clinical outcomes remain unclear.

In this retrospective cohort study, we investigated mortality and reinfection rates after surgery of patients with septic hip arthritis. We reviewed patients treated for primary septic hip joints from October 2005 to December 2016. A total of 51 adult patients were identified, and 38 among them had destructive hip joints. A poor postoperative outcome was defined as mortality or recurrent infection within 2 years of surgery.

After surgery, 7 (13.7%) patients died within 1 year and 5 (9.8%) patients developed a recurrent hip infection within 2 years. Therefore, poor outcomes occurred in 22% (n = 11) of the study cohort. Among the 38 patients with a destructive hip joint, 7 (18.4%) died within 1 year after surgery and 4 (10.5%) developed a recurrent hip infection within 2 years of surgery. Correlative infections other than infected hip joint and liver cirrhosis were identified as risk factors for poor outcomes.

In conclusion, clinical physicians treating adult primary septic hip joints should be cognizant of the high failure rate of surgical treatment. In addition, the high mortality rate should be considered during the discussion of surgical treatment with these patients and their families.

## 1 Introduction

In adult patients with hip pain, septic arthritis of the hip is a rare diagnosis.^[1,2]^ Although the hip joint is the 2nd most common joint to be affected, surgical procedures are not commonly performed for adult patients with septic hip arthritis.^[3–7]^ The study populations of primary septic hip arthritis often consist of small cohorts. In several studies, and even in a multicenter study, researchers could identify only a few adult cases^[8–22]^ with native septic hip.^[8–11]^

**Figure 1:**
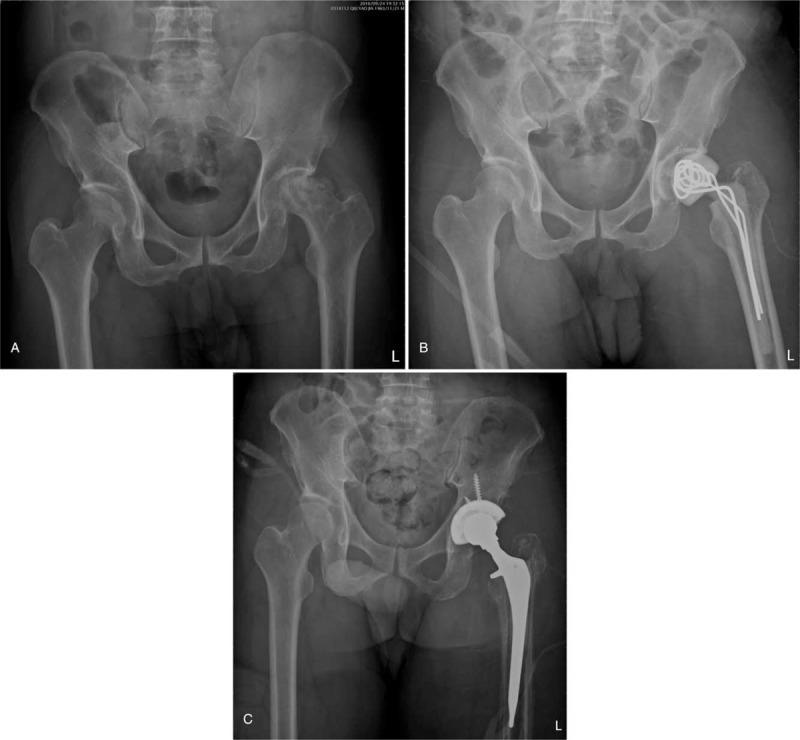
(A) A 51-year-old male patient with primary septic arthritis of the left hip joint. The hip joint was destructive with a collapsed femoral head. (B) Antibiotic-loaded cement spacer inserted in the left hip joint after resection arthroplasty. (C) Revision total-hip arthroplasty performed after controlling infection of the left hip joint.

Although rare, primary septic arthritis in adults is potentially devastating.^[10]^ The key factor in selecting the type of surgery for the primary septic hip depends mainly on symptom duration. Early onset of infection can be treated with radical open surgery or arthroscopic debridement.^[9]^ The failure rate of the surgical debridement of joints increases rapidly in the 1st few days after the onset of symptoms^[12]^; after the 1st few days, more radical surgical procedures may be needed as joint destruction (Fig. 1A) occurs. A delay beyond 3 weeks was reported to be a strong predictor of the need to sacrifice the joint.^[13]^ Hip resection arthroplasty may eradicate infection but leads to problems such as leg length discrepancy, dependency on ambulatory aids, and variable pain relief.^[14,15]^

Some authors have reported the use of antibiotic-loaded cement spacers (Fig. 1B) in the treatment of destructive coxitis.^[10,16–19]^ After controlling the infection of the primary septic hip, revision total-hip arthroplasty (Fig. 1C) can be performed for the destructive hip joint. Two-stage surgery with revision total hip arthroplasty and spacer implantation^[10,11]^ has been reported to result in a high rate of infection control but also a high mortality rate between stages. In fact, not all 2nd-stage operations for revision total hip arthroplasty are completed.^[11]^ Factors such as recurrent infection, poor patient health, and differing treatment strategies are reasons for which revision total-hip arthroplasty might not be performed. In addition, the long-term results of surgical treatment for primary septic hip are unclear. Therefore, we conducted a retrospective study to assess the failure rate of surgical treatment for the primary septic hip. We calculated the rates of mortality and recurrent infection of the affected hip joints within 2 years after surgical treatment.

## 2 Materials and methods

In this retrospective study, we reviewed medical records and X-rays from October 2005 to December 2016. Primary septic hip is defined as infection of the hip joint that developed in the absence of a fracture, insertion of an implant or prosthesis, and surgical procedure. Data acquisition and analysis in this study were approved by the Institutional Review Board of E-Da Hospital (EMRP-105-108).

We identified 55 patients with symptomatic primary septic hip joints for which surgical interventions had been performed. Surgical techniques included debridement, arthrotomy, synovectomy, resection hip arthroplasty, hip disarticulation, total-hip arthroplasty, and revision total-hip arthroplasty. Patients younger than 20 years or with a follow-up duration of <12 months were excluded. Therefore, 4 patients were excluded, and 51 patients were eligible for this study.

We recorded data regarding body mass index, smoking habits, alcohol use, number of debridement surgeries, types of microorganisms, antibiotic duration, presence or absence of destruction of the hip joint, interval between recurrent infection of the hip joint and last previous surgery, and correlative infections other than the infected hip joint (coinfection). We also used the Charlson comorbidity index to appraise baseline comorbidities.^[20]^

Patients’ clinical outcomes were recorded to define the poor postoperative outcome of primary hip joint infections. A poor postoperative outcome was considered to be when patients had recurrent hip joint infection or died within 2 years after surgery for the hip joint. Risk factors for surgical treatment of the primary septic hip were determined, and cases in which the patient received surgical disarticulation of the hip joint were excluded. To evaluate surgical treatment for primary destructive hip joint infection and the risk of surgical treatment failure, we selected patients with destructive hip joints to serve as the 2nd group.

Continuous variables are presented as mean values, whereas categorical variables are presented as numbers and proportions. A survival analysis test was performed to compare qualitative data. All statistical tests were performed using SPSS version 10.0 (SPSS, Inc, Chicago, IL). Statistical significance was set at *P* < .05.

## 3 Results

Of 51 patients with primary septic hip arthritis who received surgical treatment, 32 were men and 19 were women. The mean follow-up duration was 48.8 months. The study population included 12 patients without destructive hip joints and 39 patients with destructive hip joints. One patient with a destructive hip joint underwent hip disarticulation surgery, whereas other patients underwent resection arthroplasty (Table 1).

**Table 1:**
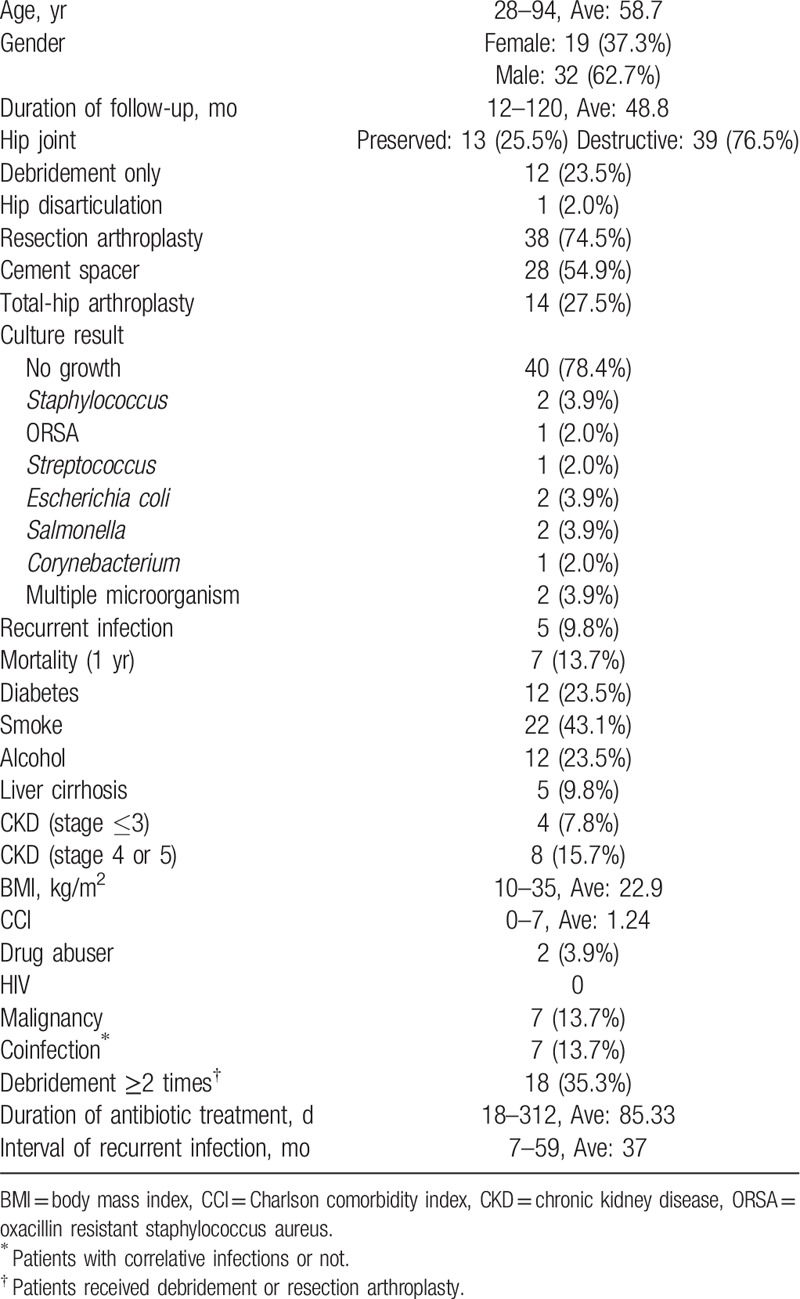
Characteristics of the 51 patients with primary septic hip.

Seven (13.7%) patients died within 1 year after undergoing surgery for the septic hip. Five patients (9.8%) had recurrent hip infections within 2 years after surgery; these patients were treated with surgical debridement. One patient died after developing a recurrent septic hip. The total poor postoperative outcome rate in this study was 22% (11 of 51 patients).

The results of the survival analysis indicated that most of the comorbidities were unrelated to a poor postoperative outcome. However, a significant difference was observed in patients with liver cirrhosis (*P* < .05), indicating that liver cirrhosis had an influence on the likelihood of having a poor postoperative outcome (Table 2).

**Table 2:**
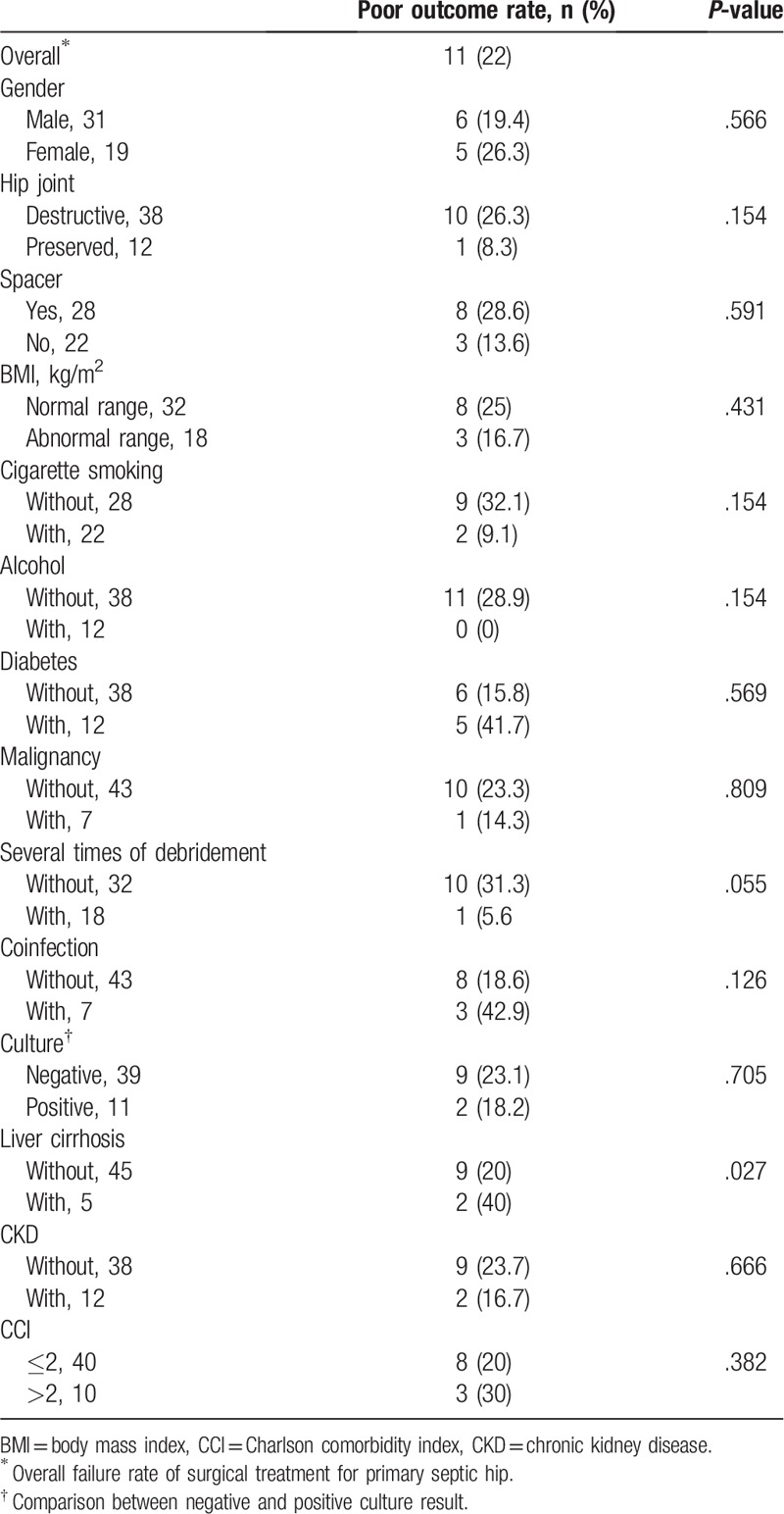
Results and risk factors for poor outcome in total 50 cases.

Of the 38 patients with primary septic arthritis of destructive hip joints who underwent resection arthroplasty surgeries, 23 were men and 15 were women. Twenty-eight (73.7%) patients had cement spacers implanted after resection arthroplasty surgery, and 14 patients underwent revision total-hip arthroplasty surgery (Table 3).

**Table 3:**
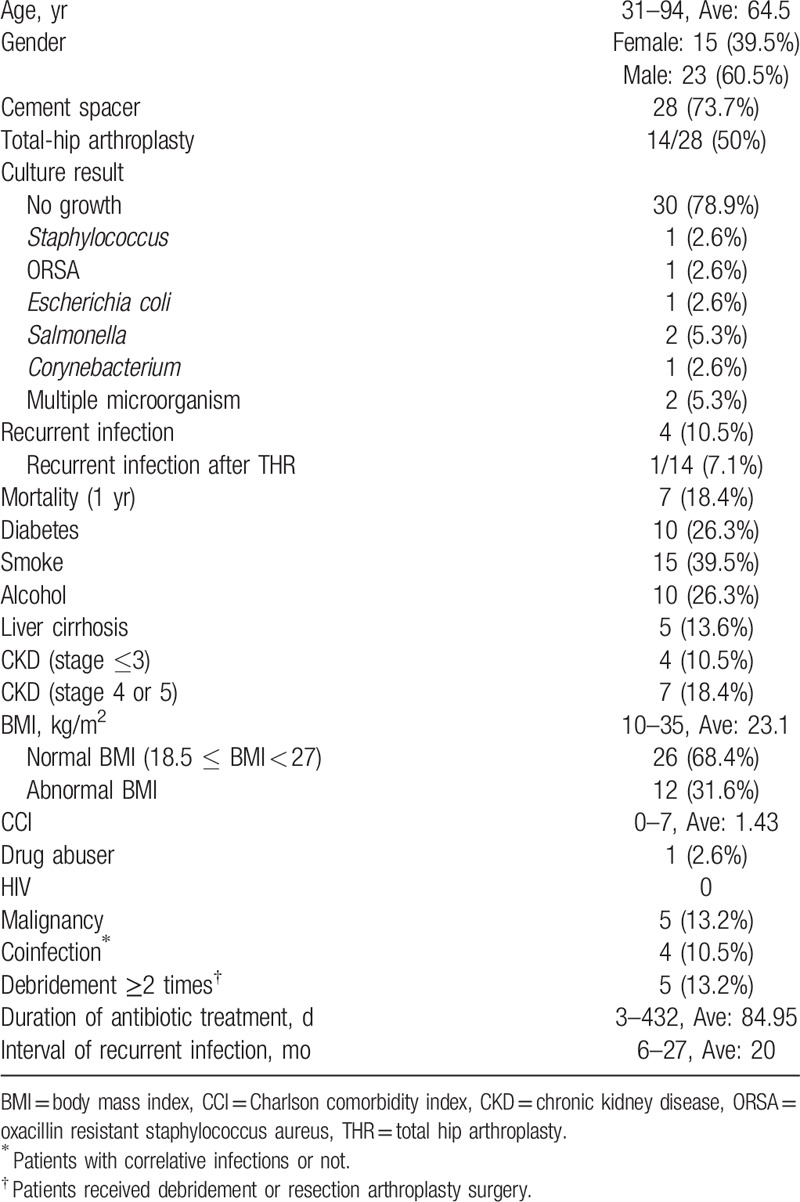
Characteristics of the 38 primary septic hip treated by resection arthroplasty.

In the resection arthroplasty group, 7 (18.4%) patients died within 1 year after undergoing surgery for the septic hip. Furthermore, 4 (10.5%) patients developed recurrent hip infections within 2 years after surgery; these patients were treated with surgical debridement. The total poor postoperative outcome rate of the resection arthroplasty group was 26.3% (10 of 38 patients).

Of the 14 patients who underwent revision total-hip arthroplasty, none died and only one developed a recurrent infection. The success rate of patients who underwent revision total-hip arthroplasty was 92.9% (13 of 14 patients).

The results of the survival analysis test revealed that patients who underwent several debridement surgeries for the septic hip joint had a low rate of unfavorable postoperative outcomes after undergoing surgical treatment for primary septic arthritis of destructive hip joints (Table 4). Furthermore, the results revealed a significant difference in the postoperative outcomes of patients with coinfection (i.e., infections other than in the hip joints, *P* *<* .05), indicating that coinfection was related to unfavorable postoperative outcomes.

**Table 4:**
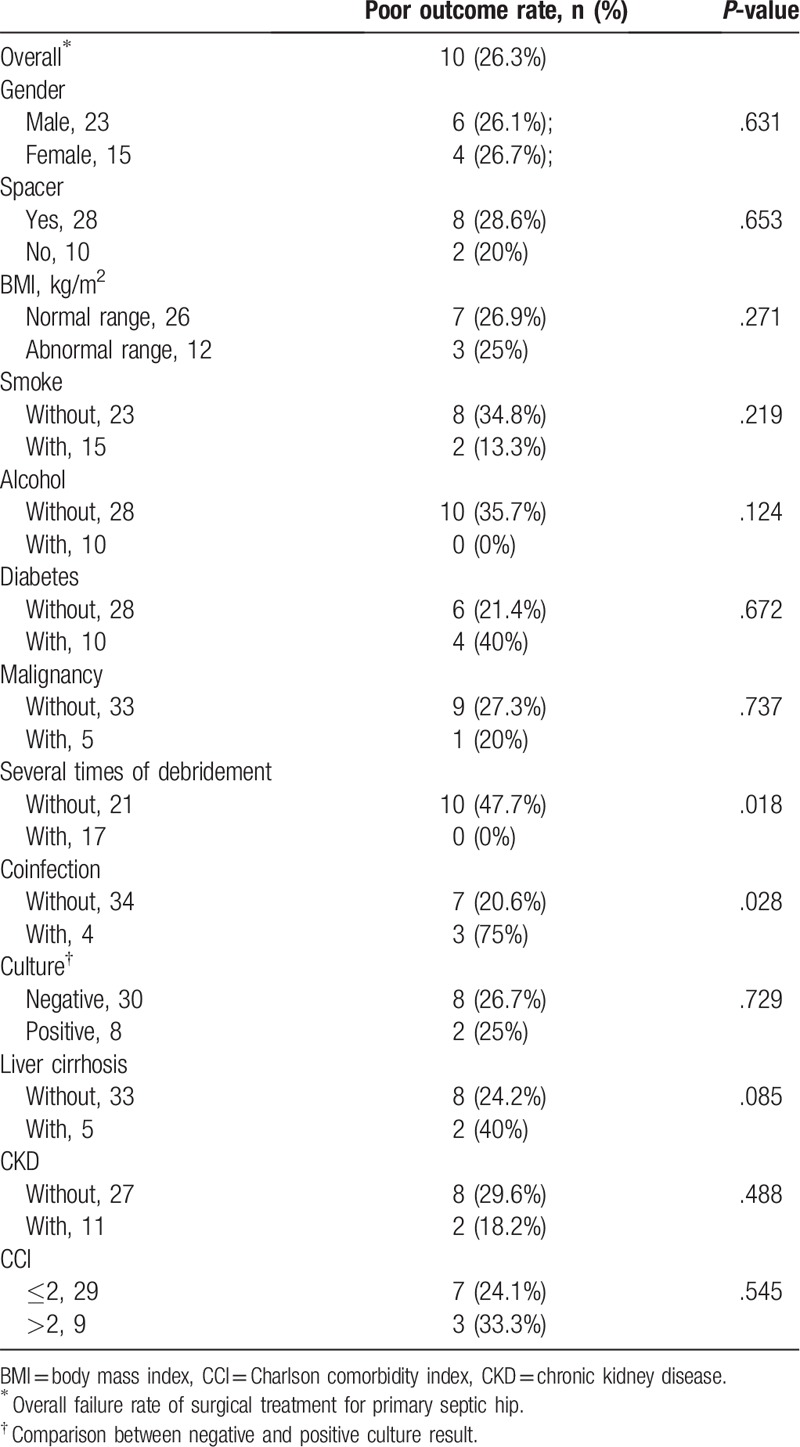
Results and risk factors for poor outcome.

## 4 Discussion

Adult primary septic arthritis is a potentially devastating disease.^[10]^ The mortality rate after the 1st stage was reported to be 18% in a 2-stage protocol with spacer implantation for destructive hip joints.^[10]^ In the present study, the 1-year mortality rate after surgery was 13.7%. In addition, that in the destructive septic hip group was even higher (18.4%). Therefore, when clinical physicians encounter adult patients with primary septic arthritis of hip joints, they must be aware of this high mortality rate, even when aggressive surgical treatments are performed for these patients.

The use of antibiotic-loaded hip spacers might be considered to be a suitable method for managing such primary hip joint infections.^[10,11,16–19,21–23]^ A 2-stage protocol with spacer implantation for adult primary septic arthritis of hip joints could provide effective infection control.^[10,11]^ Physicians typically have 2 goals when treating primary septic arthritis of destructive hip joints: the main goal is infection control, and the other goal is reconstruction of the hip joint. For treating infection of primary septic hip joints, surgery, and adequate antibiotics are essential. However, various surgical procedures can be applied. Resection arthroplasty can control infection,^[11]^ but leg length discrepancy is a potential problem. Although hip arthroplasty with hip prosthesis can be used to restore hip function, a hip prosthesis is a foreign body part that has no power to resist infection and may result in a high periprosthetic infection rate.^[24]^ The 2nd-stage procedure of hip arthroplasty must be performed after satisfactory infection control. In our study, we found that only 50% of patients (14 of 28) received 2nd -stage surgery for hip arthroplasty after resection arthroplasty for infection control.

The most commonly detected pathogens for septic hip joints unrelated to injuries or medical interventions are *Staphylococcus aureus* and *Streptococcus pyogenes*.^[6,25,26]^ However, 78.4% of the culture results in our study did not have identical pathogens Therefore, empirical antibiotics were most commonly used in our study. Prolonged systemic antibiosis might be used to control joint infection as well as prevent further septic metastases.^[11]^ The average duration of antibiotic therapy in our study was 84.95 days, and the recurrent infection rate in septic destructive hip joints was only 10.5%.

No standard antibiotic regimen was used in our study. Lausmann et al^[27]^ reported promising preliminary results of septic revision arthroplasty that was performed using a combination of clindamycin and gentamicin in the bone cement. This procedure was associated with a high success rate for eradication of infection at stage one of septic exchange as well as for prevention of infection in high-risk patients, such as those with comorbidities. Lausmann et al used the ENDO spacer technique, which involves the use of a dual-mobility liner and a downsized stainless cemented straight stem in combination with the antibiotic-loaded bone cement to preserve hip motion. This standardized technique can serve as a potential method to treat primary septic arthritis of hip joints without identified pathogens. In addition, ozone therapy in conjunction with antibiotics was reported to successfully treat cases of septic prosthetic joints.^[28]^ These treatment strategies can be considered in the future to treat challenging cases of primary septic arthritis of the hip joints.

Previous reports regarding complications of primary septic hips have been related to chronic osteomyelitis, extra-articular abscess formation, pathologic dislocation, and sepsis. Additionally, mortality rates have been reported for elderly, multimorbid, or immunosuppressed patients.^[2,29]^ To address the modifiable and nonmodifiable factors associated with the risk of revision for periprosthetic joint infection after primary hip arthroplasty, Lenguerrand et al^[30]^ recommended the following strategies: for modifiable factors, identification of modifiable factors, use of targeted interventions, and beneficial modulation of modifiable factors are recommended, and for nonmodifiable factors and factors that exhibit time-specific effects, appropriate counseling of patients preoperatively should be considered by clinicians. In our study, we evaluated some comorbidities associated with treatment failure and discovered that coinfection and liver cirrhosis might have contributed to failure outcomes. In the future, the treatment strategy for patients with primary septic hips who have these risk factors might be modified to treat the coinfection 1st along with some adjuvant therapy, such as ozone therapy, before surgery. In our study, patients who received surgical debridement several times for the septic hip joint appeared to yield favorable clinical results. Our results differed from those reported by Hunter et al, who indicated that most septic joints could be managed with single debridement.^[31]^ According to our study, performing surgical debridement several times for the primary septic hip joint is effective and should always be considered for cases with poor infection control.

Finally, our study has the following limitations. First, this retrospective study included a small number of patients, which may have led to statistically nonsignificant results. Second, the study population was recruited from only one hospital; thus, the exact prevalence of adult primary septic hip joints in the general population could not be determined.

In conclusion, clinical physicians should be cognizant of the high failure rate of surgical treatment for adult primary septic hip joints. Furthermore, in consideration of the high mortality rate, surgical treatment should be discussed with these patients and their families.

## Acknowledgment

The authors thank Ms Chen Tzu-Shan for her efficient assistance. This manuscript was edited by Wallace Academic Editing.

## Author contributions

**Conceptualization:** Yao-Chun Hsu, I-Ming Jou.

**Validation:** Pao-Hsin Liu, I-Ming Jou.

**Writing – original draft:** Feng-Chen Kao.

**Writing – review & editing:** Yao-Chun Hsu, Yuan-Kun Tu.

FengChen Kao orcid: 0000-0002-6068-3144.
